# Transitions in symbiosis: evidence for environmental acquisition and social transmission within a clade of heritable symbionts

**DOI:** 10.1038/s41396-021-00977-z

**Published:** 2021-05-03

**Authors:** Georgia C. Drew, Giles E. Budge, Crystal L. Frost, Peter Neumann, Stefanos Siozios, Orlando Yañez, Gregory D. D. Hurst

**Affiliations:** 1 Department of Zoology, University of Oxford, Oxford, UK; 2 Institute of Infection, Veterinary and Ecological Sciences, University of Liverpool, Liverpool, UK; 3 School of Natural and Environmental Sciences, Newcastle University, Newcastle upon Tyne, UK; 4 Institute of Bee Health, Vetsuisse Faculty, University of Bern, Bern, Switzerland

**Keywords:** Microbial ecology, Molecular evolution, Bacterial evolution, Bacterial genetics, Phylogenetics

## Abstract

A dynamic continuum exists from free-living environmental microbes to strict host-associated symbionts that are vertically inherited. However, knowledge of the forces that drive transitions in symbiotic lifestyle and transmission mode is lacking. *Arsenophonus* is a diverse clade of bacterial symbionts, comprising reproductive parasites to coevolving obligate mutualists, in which the predominant mode of transmission is vertical. We describe a symbiosis between a member of the genus *Arsenophonus* and the Western honey bee. The symbiont shares common genomic and predicted metabolic properties with the male-killing symbiont *Arsenophonus nasoniae*, however we present multiple lines of evidence that the bee *Arsenophonus* deviates from a heritable model of transmission. Field sampling uncovered spatial and seasonal dynamics in symbiont prevalence, and rapid infection loss events were observed in field colonies and laboratory individuals. Fluorescent in situ hybridisation showed *Arsenophonus* localised in the gut, and detection was rare in screens of early honey bee life stages. We directly show horizontal transmission of *Arsenophonus* between bees under varying social conditions. We conclude that honey bees acquire *Arsenophonus* through a combination of environmental exposure and social contacts. These findings uncover a key link in the *Arsenophonus* clades trajectory from free-living ancestral life to obligate mutualism, and provide a foundation for studying transitions in symbiotic lifestyle.

## Introduction

Microbes that associate with hosts span a continuum from mutualism to parasitism and employ disparate transmission strategies [1]. Heritable bacterial symbionts, such as *Wolbachia* and *Arsenophonus*, transmit vertically (VT) from parent to offspring [2]. Other microbial symbioses form via horizontal transmission (HT) during host development, by acquisition from environmental reservoirs or via infected conspecifics or other host taxa [3, 4]. In some cases, a combination of these routes operate, creating a complex transmission landscape [5, 6]. Across this transmission axis lies the impact of the symbiosis on each partner—whether the host and microbe benefit from the interaction, and whether one party requires the other to complete their life cycle. Symbioses vary from being facultative (where one partner does not require the other) to obligate (where there is dependence). Obligacy may be a component of symbiont or host biology, or in some cases both parties are mutually dependent [2, 7, 8].

Clades of heritable symbionts commonly include strains that are both facultative and obligate from the host perspective. In contrast, for the microbe, life is commonly obligately symbiotic, generally lacking replicative or dormant phases outside of host organisms [7]. Despite this dependence, many of these symbionts can also transmit horizontally (inter and intra-specifically) over evolutionary timescales [9–11]. However, it is generally accepted that VT predominantly drives population dynamics [12], but see [13]. In contrast, exclusively horizontally acquired symbionts must be transmitted to new hosts via infected con-/hetero-specifics or environmental reservoirs [14, 15]. As a result, symbiotic life for these microbes is commonly facultative [3]. The absence of VT can promote higher rates of partner switching [2, 16] and a weaker association between host and symbiont fitness. Consequently, selection for higher virulence and trajectories toward parasitism are commonly assumed to be favoured more readily in HT symbionts [1, 17, 18], but see [19, 20].

Symbioses thus exist on an evolutionary landscape in which transitions between transmission mode and lifestyles occur. However, study of the drivers and impact of transitions in transmission mode is inhibited by a lack of clades in which different lifestyles co-occur among members. Clades encapsulating diverse transmission biology enable understanding of both the ecological and evolutionary mechanisms driving transitions, and the consequences for processes such as genome evolution [21–24]. For instance, the emergence of a horizontally transmitted vertebrate pathogen (*Coxiella burnetti*) from a clade of maternally inherited tick endosymbionts [22] provides insight into how infectious transmission can emerge from a heritable, obligate clade. Likewise, the presence of an opportunistic human infective *Sodalis* provides an important comparator for understanding the evolution of symbiosis, through comparison to insect-associated mutualistic lineages of *Sodalis* [21].

As a monophyletic clade of heritable Enterobacteriaceae, *Arsenophonus* provides a valuable base for exploring the evolution of a heritable lifestyle [25], due to its wide host distribution (est. 5% of arthropod species) [26] and diversity in symbiotic lifestyle [27]. Members of the clade include reproductive parasites [28], facultative mutualists [29], and highly coevolved obligate endosymbionts undergoing genomic decay [30–33]. Despite this diversity, all strains characterised to date are VT transmitted. While frequent HT over ecological timescales has been shown for a number of strains, including the male-killer *Arsenophonus nasoniae* [13, 34, 35] and two species of insect-vectored phytopathogens (*Arsenophonus phytopathogenicus* and *Phlomobacter fragariae*) [36–38], this route occurs alongside vertical transmission. As a result, *Arsenophonus* is commonly reported from arthropod screening efforts and presumed heritable without further characterisation [26].

An economically important eusocial host, the Western honey bee (*Apis mellifera*), has previously been associated with *Arsenophonus* [39–43] and infection has been linked to poor health outcomes [44] including colony collapse disorder [45]. Whilst this interaction has attracted interest from the community, basic information on the epidemiology and transmission of *Arsenophonus* in honey bee populations is lacking. Symbionts in eusocial hosts are exposed to markedly different selection pressures from those in solitary species. This difference arises from a higher density of hosts, greater host relatedness and a homoeostatic nest environment, as well as reproductive division of labour, overlapping generations and cooperative brood care [46, 47]. Specialised social behaviours [48] additionally foster the transmission of microbes by direct contact, such as via proctodeal (anus—mouth feeding) and stomodeal trophallaxis (mouth—mouth feeding) [49]. Thus, host sociality is an important driver of symbiont phenotypes [47, 49, 50], and indeed interesting heterogeneities in symbiont infections are emerging based on caste, sex [51–55] and degree of sociality [50]. Despite this, the potential effects of host sociality on symbiont ecology and evolution, including important phenotypes such as transmission, remain largely unexplored.

This study focuses on characterising the ecology and transmission of *Arsenophonus* from a eusocial host. We use a phylogenomic approach to show robust placement of the strain within the *Arsenophonus* clade and genomic analysis to report on its predicted metabolic properties and genome features. We then present data tracking the prevalence of the symbiont in honey bee colonies over space and time, to search for indicators of stable maintenance (implying VT) or changes in prevalence (implying infectious transmission epidemics). These data are complemented by fluorescence in situ hybridisation (FISH) analysis highlighting tissue interactions with honey bees. Finally, the capacity for vertical transmission is assessed directly by screening for *Arsenophonus* across host life history, and the ability to horizontally transmit intra-specifically is investigated under differing social conditions. These data demonstrate the presence of an infectiously transmitting *Arsenophonus* without vertical transmission, highlighting transitions in life history within this important genus of insect-associated microbes.

## Methods

### Phylogenomic position of *Arsenophonus* from honey bees and comparative genomic analysis

To assess the relatedness of the *Arsenophonus* strain associated with honey bees to other *Arsenophonus*, a phylogenomic approach was adopted. To this end, a draft genome was assembled (bioproject accession: PRJEB39047) using Illumina paired end shotgun library reads obtained in a previous study derived from haemolymph template purified through a Nycodenz gradient (see Gauthier et al. [56]). The assembled contigs were annotated using prokka v1.14.0 [57] and completeness was assessed using BUSCO v4.1.4 based on the presence of 124 universal bacterial marker genes [58]. The phylogenetic position of the *Arsenophonus* strain was estimated in relation to other *Arsenophonus*, with the free-living species *Proteus mirabilis* and *Providencia stuartii* used as outgroups, using first an alignment of 53 ribosomal proteins and then a concatenated set of 155 single copy core orthologus proteins. In addition, we compared the predicted metabolic potential of *Arsenophonus* from bees to other strains, and in greater detail examined functional differences and the degree of synteny to *A. nasoniae* (see Supplementary information for details).

### Spatial and seasonal dynamics of *Arsenophonus* in honey bee colonies

To monitor *Arsenophonus* prevalence over time and space, adult workers were collected from the outer frames of 159 colonies stemming from 45 apiaries in ten counties across England. Colonies were sampled from April to November during 2014–2018, and repeated screening of some colonies resulted in a total of 230 sampling events. Apiaries are defined here as a collection of colonies that are colocalised within a small area. Bees were preserved in 70% EtOH at −20 °C until DNA extraction. For each colony, posterior legs were pulled from workers (*n* = 12), pooled in groups of four and exposed to UV light for 10 min to cross link DNA from surface microbes. Legs were used as this tissue is a reliable marker of *Arsenophonus* association in bees and did not inhibit downstream PCR assays when DNA was extracted using a high throughput Chelex protocol [59]. Extraction quality was verified by amplification of host DNA (EF1-α) [60] and the presence of *Arsenophonus* spp. established by PCR assays targeting *fbaA* (adapted from Duron et al. [34], see Supplementary information) and Sanger sequencing of the product to determine broad identity of the *Arsenophonus* strains. Sensitivity of PCR assays was established through serial dilution, and was robust over two orders of magnitude (see Supplementary information).

To determine if *Arsenophonus* is lost from honey bee colonies during overwintering, the status of 25 colonies (from seven apiaries) was tracked from autumn to spring at a greater depth than described above. In the autumn, 15 worker bees were screened from each colony (three individuals pooled per extraction) to determine *Arsenophonus* prevalence. If 80% of extractions (i.e. 4 of 5) were positive for *Arsenophonus* the colony was included in the infected cohort (group A, *n* = 19). The uninfected cohort (group B, *n* = 6) comprised of colonies where all extractions returned negative. Colonies were left to overwinter, and the sampling process was repeated in the spring for colonies that overwintered successfully. To determine infection status in spring a total of 24 bees were screened per colony (eight pools of three bees). DNA was extracted from whole bees (heads removed prior to extraction to minimise PCR inhibition [61]) by Promega Wizard purification, with PCR detection of *Arsenophonus* (see Supplementary information).

### Localisation of *Arsenophonus* within the gut

To visualise *Arsenophonus* using FISH, whole guts were dissected from live worker bees and placed into Carnoy’s fixative for 24–48 h, washed with 100% EtOH (×3) and incubated with hybridisation buffer at room temperature in the dark (~15 h, see Supplementary information for detail). The symbiont was targeted using an *Arsenophonus* specific probe (TCATGACCACAACCTCCA) [62] with a 5′ Alexa Fluor 647 fluorochrome. Tissues were washed with pre-heated buffer (∼48 °C, ×3 for 10 min) and mounted on glass slides with DAPI counter-staining. Tissues cured for 24 h before visualisation by confocal microscopy (ZEISS LSM 88, ×40 objective lens), after which multiple optical sections were assembled into Z-stacks under maximum intensity settings using ImageJ [63]. Gut tissue of bees from colonies not associated with *Arsenophonus* underwent the same process to function as negative controls (see Supplementary information). In addition to gut imaging, faecal samples were collected from workers (*n* = 22) isolated in sterile petri dishes and extracted by Promega Wizard purification (see Supplementary information). Material was then tested for *Arsenophonus* DNA through PCR assay.

### Assessing the heritability of *Arsenophonus* and acquisition from infected conspecifics

To determine heritability of *Arsenophonus* in honey bees, infection was assessed across life history. Frames of worker brood were removed from managed field colonies (*N* = 8, A+ = 6, A− = 2) where *Arsenophonus* status had previously been determined with high confidence. Eggs (*n* = 29), larvae (*n* = 67), pupae (*n* = 49), newly emerged workers (NEWs) (*n* = 36), adult workers (*n* = 45) and where possible drones (*n* = 22) were collected concurrently. To asses if *Arsenophonus* association is heritable but only becomes detectable later in the life history, a further 64 NEWs were removed from colonies and left to develop to forager age (25 ± 2 days post-emergence) in the company of other NEWs only. DNA was extracted individually from eggs and early stage larvae using a Qiagen DNeasy Blood & Tissue Kit, for all other life stages Promega Wizard purification was used. Molecular detection was completed as previously.

To assess if infections were maintained in the absence of the colony and foraging environment, infected individuals were detained in the laboratory and their *Arsenophonus* status tracked over 15 days. Adult workers were collected from two colonies (total *N* = 76, Colony A = 36, Colony B = 40) with a high prevalence of *Arsenophonus* in 15 workers (>90%) or one control colony where none of 15 workers tested positive (*N* = 32) (based on *Arsenophonus* detection of individual bees, see Supplementary information). On entering the laboratory (day 1) the *Arsenophonus* status of all individuals was established by removal of posterior tarsus tissue (‘leg snip’) and PCR assays for symbiont presence (see Supplementary information). Additional bees (‘no leg snip control’, *N* = 40) from the infected source colonies were not subjected to leg snips to establish if this method biased results. Individuals were marked and maintained in groups of four. To track the maintenance of *Arsenophonus* over time, individuals were randomly selected from each group and culled at days 4, 8 and 15. Individual *Arsenophonus* status was determined using the same methodology as day 1, but using opposing tarsus tissue.

To assess the capacity for horizontal acquisition of the symbiont, adult workers (donors) were taken from colonies (*N* = 6) with a high prevalence of *Arsenophonus* in 15 adult bees (>85% infected, see Supplementary information) and mixed in 340 ml pots with uninfected NEWs (recipients). Each pot contained ten donors and five recipients. Two transmission treatments were established, each with ten replicates. In the ‘general contact’ treatment, bees were allowed to freely contact one another within the pot and food (50% sucrose solution) was openly available. In the ‘trophallaxis’ treatment, recipients with no direct access to food were separated from donors (with food) by fine mesh, forcing infected bees to feed uninfected individuals by trophallaxis, but preventing other social contact. Contacts were allowed for 5 days, after which the fate (dead or alive) of all individuals was recorded, i.e. those that had died during the course of the experiment (prior to final cull) were labelled dead and analysed separately. In each case, experiments were compared to controls in which uninfected bees were mixed with recipient bees. DNA was extracted individually from whole bees (test; *n* = 300, control; *n* = 165) by Promega Wizard purification.

### Statistical methods

For statistical analyses, generalised linear models (GLM) and mixed models (GLMM) were fitted in R version 3.3.1 [64] with binomial error distributions and logit link function, using the packages *glm* and *lme4* [65]. Minimum adequate models were selected by Akaike information criteria [66, 67] and likelihood ratio tests (LRT), with the latter being used to assess the significance of fixed and random effects [68]. Overdispersion was assessed using the Blemco package [64]. See Supplementary information tables detailing selection of statistical models.

## Results

### Phylogenomic position of the honey bee *Arsenophonus* in the wider clade and metabolic competence

**Fig. 1:**
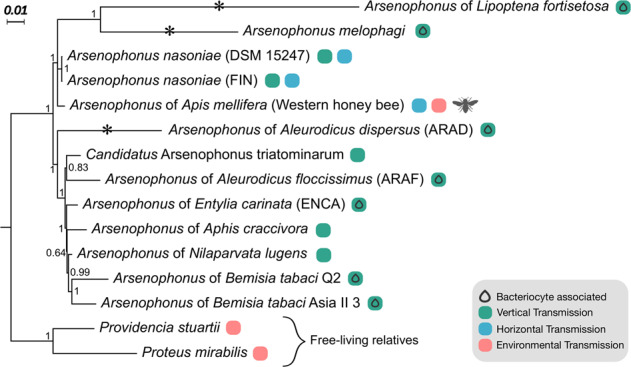
Transmission mode and lifestyle diversity in the *Arsenophonus* clade. Phylogenomic position of the *Arsenophonus* associated with honey bees (this study) among *Arsenophonus* spp. with available genomes. Bacterial species names are given, if formally recognised, else the associated insect host is given. Symbols indicate known bacteriocyte associations and transmission modes, vertical transmission (green), horizontal transmission (blue), if an environmental transmission route is additionally inferred this is indicated in pink. Analysis based on 53 ribosomal proteins support for Bayesian inference (posterior probabilities) is shown at nodes (Color figure online).

Phylogenomic analysis based on 53 ribosomal protein sequences unambiguously placed the honey bee associated strain within the *Arsenophonus* clade (Fig. 1) alongside known heritable symbionts. The strain was established as a sister strain, with strong support, to *Arsenophonus* strains infecting other hymenopteran hosts (parasitoid wasps), including the male-killing reproductive parasite *A. nasoniae*. These results were mirrored in analysis of a wider set of core genes, in which the *Arsenophonus* from honey bees again clustered as sister to *A. nasoniae* (Supplementary information Fig. 1).

**Fig. 2:**
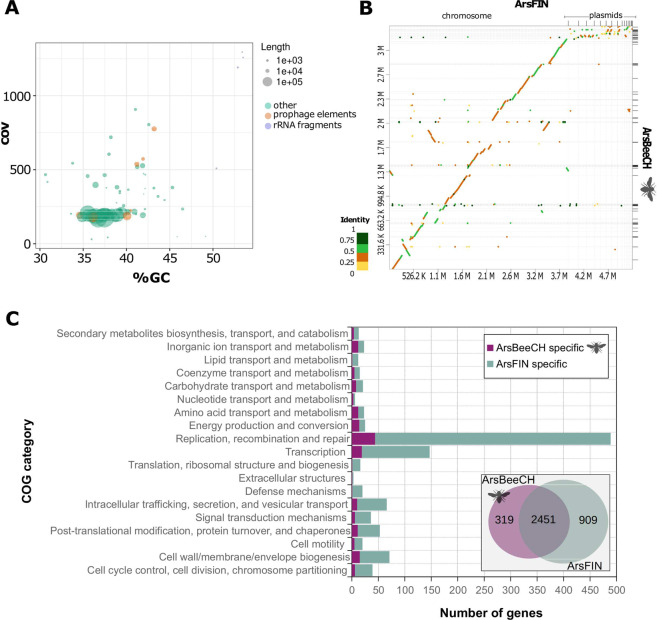
Overview of the genome assembly of *Arsenophonus* from honey bees (ArsBeeCH) and comparison with close relative *Arsenophonus nasoniae* (ArsFIN). **A** GC% vs coverage plot of the ArsBeeCH draft assembly. Contigs with putative phage origin were identified using PHASTER web server based on sequence similarities searches. **B** Syntenic comparison between ArsBeeCH and ArsFIN genomes as determined by D-Geneis and minimap2. **C** COG functional annotation of strain-specific genes. The inset Venn diagram shows the number of shared and unique ortholog groups between the two *Arsenophous* strains.

We assembled a draft genome for *Arsenophonus* from bees and examined synteny and compared gene content to *A. nasoniae* (Fig. 2), which is a closed completed genome [69]. At 3.3 Mb, the draft genome was larger than most other sequenced *Arsenophonus* with the exception of *A. nasoniae* and *Candidatus* Arsenophonus triatominarum (Supplementary information Table 1). BUSCO completeness score for the bee *Arsenophonus* strain mirrored that of *A. nasoniae* (C:99.2% [S:99.2%, D:0.0%], F:0.8%, M:0.0%, n:124) (Supplementary information Fig. 2) suggesting a near complete genome. Synteny analysis supported the close relationship of the two strains, with evidence of some structural differences including at least one inversion event and several indels (Fig. 2). As expected, accessory elements (plasmids) were less well conserved between the two genomes. Overall, 2451 orthologous groups of proteins were shared between the two symbionts, with 319 found solely in *Arsenophonus* from bees, and 909 solely in *A. nasoniae*. This higher number of unique orthologous genes in *A. nasoniae* may reflect the difference between draft and polished genomes, particularly in accessory genes carried on plasmids and prophage elements.

*Arsenophonus* from bees also clustered with *A. nasoniae* in analysis of predicted metabolic potential (Supplementary information Figs. 3 and 4). These strains shared higher predicted competency for the glyoxylate cycle, polyamine biosynthesis compared to other sequenced *Arsenophonus*, and reduced capacity for cysteine biosynthesis. Predicted differences in metabolic capacity between *Arsenophonus* from bees and *A. nasoniae* were modest, making similar metabolic potential and culture requirements likely.

### Spatial and seasonal dynamics of *Arsenophonus* in honey bee colonies

**Fig. 3:**
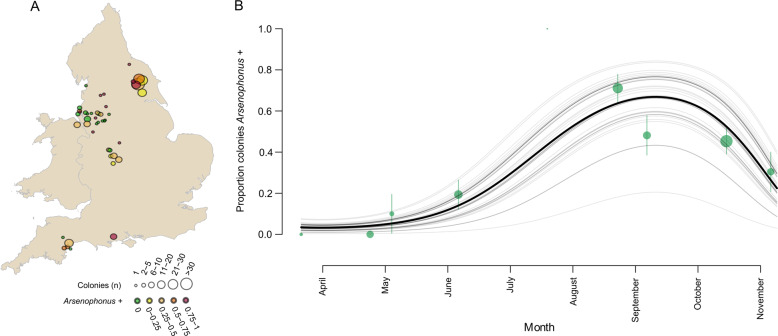
Spatial and seasonal dynamics in the *Arsenophonus*–honey bee association. **A** Circles show the approximate locations of 45 honey bee apiaries (colonies colocalised within a small area) sampled for *Arsenophonus* across England (2014–2018). Circle size reflects the number of colonies sampled within an apiary location and colour indicates the proportion of colonies associated with *Arsenophonus*. **B** The proportion of colonies testing positive for *Arsenophonus* from spring to autumn was modelled using 229 binomial observations of *Arsenophonus* status from 159 colonies (2014–2018). An overall prediction for all apiaries (black) and individual predictions for each apiary (grey, *n* = 45) are shown. Green circles represent the mean prevalence of *Arsenophonus* by month at the mean monthly sampling date, size is proportional to the square root of the number of colony samplings. Error bars represent binomial CI (Color figure online).

*Arsenophonus* was found associated with 38.9% (95% CI: 32.5–45.6%, *N* = 159 colonies) of honey bee colonies across the UK. Infections were spatially widespread and identifiable in all ten county regions tested (Fig. 3A). County region was not a significant predictor of *Arsenophonus* infection (GLMM, LRT: *X*^2^ = 0, df = 1, *p* = 1), but spatial patterns were evident at a local scale, with apiary emerging as an important covariate, with colonies within an apiary having correlated infection status (GLMM, LRT: *X*^2^ = 13.8, df = 1, *p* = 0.002**). All *Arsenophonus* detected had identical sequence at the *fbaA* locus (*N* = 159).

Strong seasonal dynamics in *Arsenophonus* infections (Fig. 3B) were evident, with prevalence of the bacterium changing in a non-linear fashion during the main foraging period of the host (March to October). *Arsenophonus* prevalence is lowest during the spring (2.63% of colonies, 95% CI: 0.0670–13.8%, *N* = 38) with the first infected colony detected in May. Prevalence continues to rise, reaching a peak in late summer (August: 71% of colonies, 95% CI: 55.7–83.6, *N* = 45), before dropping into Autumn (43.5% of colonies, 95% CI: 34.3–53.0, *N* = 115). Significant temporal variation by year was observed for *Arsenophonus* prevalence (GLMM, LRT: *X*^2^ = 3.87, df = 1, *p* = 0.049*). See Supplementary information Table 2 for a full summary.

**Fig. 4:**
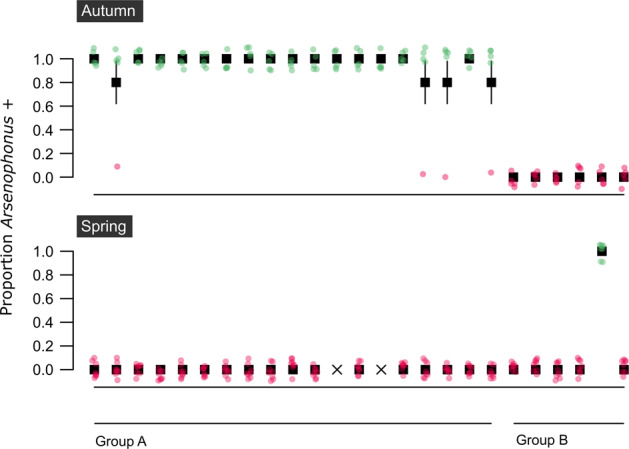
Overwintering loss of *Arsenophonus* at a colony level. Group A colonies (*n* = 19) were infected with *Arsenophonus* in autumn but the bacterium was undetectable by the following spring. Group B colonies (*n* = 6) were uninfected with *Arsenophonus* in autumn, one colony gained *Arsenophonus* association by spring. Colonies that did not survive the winter (*n* = 2) are denoted by an X. The proportion of bees testing positive for *Arsenophonus* in each colony is shown. Note, fewer bees were sampled in Autumn (*n* = 15 bees per colony, pooled in groups of 3) than spring (*n* = 25 bees per colony, pooled in groups of 3). Pink dots (0 = *Arsenophonus−*) and green dots (1 = *Arsenophonus*+) show the raw binomial data jittered. Error bars indicate binomial SE (Color figure online).

In temperate environments, overwintering conditions represent a distinct state for honey bees, with foraging ceasing temporarily and survival dependent on stored colony resources. The *Arsenophonus* infection status of 25 honey bee colonies was tracked from autumn to spring (Fig. 4). Of the 23 colonies that survived winter, all of those infected with *Arsenophonus* in the autumn (Group A, *n* = 17) had lost the bacterium by spring. Of the colonies where *Arsenophonus* was not detected in the autumn (Group B, *n* = 6), five remained uninfected in spring, and one case gained *Arsenophonus*. Confidence in this newly positive colony is high, as all pools tested (*N* = 8 pools of three bees) were positive for *Arsenophonus*. Notably, this colony was sampled latest in the spring season.

### Localisation of *Arsenophonus* within the gut

**Fig. 5:**
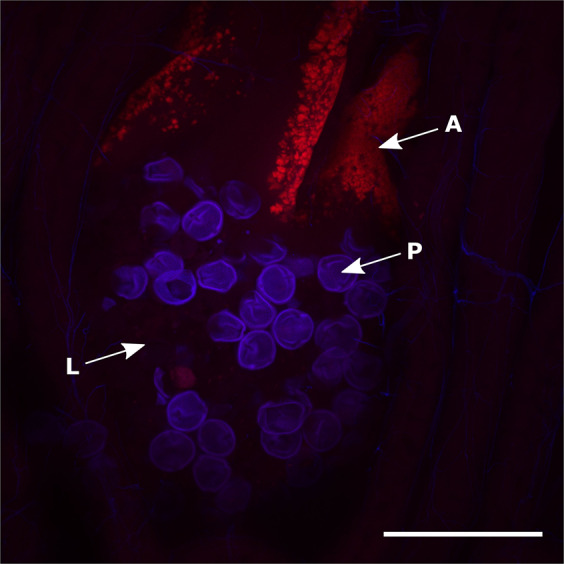
Localisation of *Arsenophonus* bacteria in the midgut of a worker honey bee. Confocal microscopic image of a whole honey bee gut mount with an *Arsenophonus* specific Alexa Fluor^®^ 647 labelled probe (red fluorescence) and DAPI counter-staining (blue fluorescence). Hybridisation of an *Arsenophonus* specific probe (A) is visible within the lumen (L) of the midgut (ventriculus) alongside pollen grains (P). The image is a composite Z-stack comprised of 32 optical slices (ZEISS LSM 880) assembled in ImageJ under maximum intensity, scale bar represents 100 μm. Amplicon based approaches also report *Arsenophonus* from haemolymph [56]. For confocal images of whole gut mounts from honey bees uninfected with *Arsenophonus* see Supplementary information Fig. 5 (Color figure online).

Previous work using metagenomic analysis has reported *Arsenophonus* in the gut [40, 41], on the cuticular surface [39] and in haemolymph [56]. To obtain confirmation of symbiosis, targeted imaging of gut tissue from infected honey bees was conducted. This analysis showed aggregations of *Arsenophonus* (coloured red) in the midgut (Fig. 5) sufficiently large to imply colonisation and replication. More sporadic infections were apparent in the crop, and also the rectum (consistent with low representation of *Arsenophonus* in honey bee gut microbiome studies that focus on the hindgut). Control images of uninfected bee tissue suggested neither autofluorescence nor inadequate probe removal contributed to artefactual *Arsenophonus* visualisation. The absence of a strong signal in the rectum is consistent with specific binding, as there was no widespread probe binding in the rectum of *Arsenophonus* positive individuals (see Supplementary information Fig. 5). *Arsenophonus* was also detected by PCR assay in 59.1% of faeces from infected bees (95% CI: 36.4–79.3) suggesting the bacterium may be shed from the gut, however viability of the symbiont was not confirmed.

### Assessing the heritability of *Arsenophonus* and acquisition from infected conspecifics

**Fig. 6:**
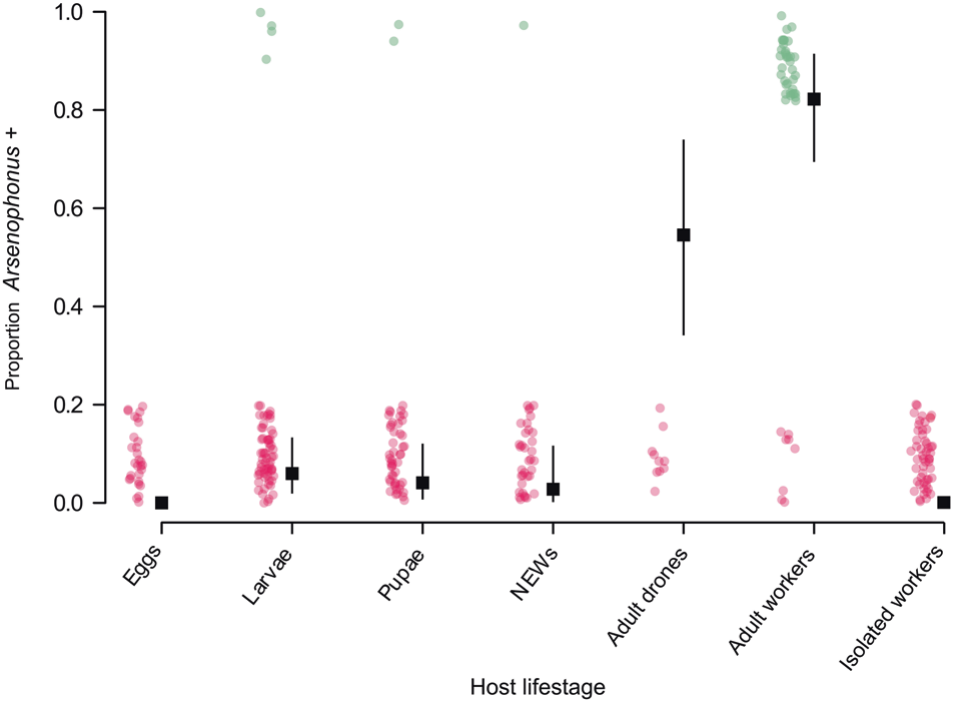
Vertical transmission is largely absent in the *Arsenophonus*–honey bee association. Samples from across the host life history were taken from colonies (*n* = 6) identified as *Arsenophonus* positive (based on workers) and screened for *Arsenophonus*. Eggs (*n* = 29), larvae (*n* = 67), pupae (*n* = 49) and newly emerged workers (NEWs) (*n* = 36) were all of worker caste. Isolated workers (*n* = 45) were removed from colonies as NEWs and allowed access only to other NEWs before screening for *Arsenophonus* at forager age (25 days ± 2). Binomial GLM predictions for the proportion of each life-stage infected are shown with 95% CI. Pink dots (0 = *Arsenophonus−*) and green dots (1 = *Arsenophonus*+) show the raw binomial data jittered (Color figure online).

To date, all characterised *Arsenophonus* strains show vertical transmission in their arthropod hosts. To assess if the honey bee strain conforms to this transmission strategy, the presence of *Arsenophonus* was assessed across host life history (Fig. 6). There was no evidence of transovarial transmission of *Arsenophonus* with eggs consistently testing negative, corroborating previous findings in honey bees [43]. In other early life stages *Arsenophonus* was detected only at low frequencies, with 5.97% of larvae (95% CI: 1.65–14.6, *p* < 0.001***), 4.081% of pupae (95% CI: 0.498–14.0, *p* < 0.001***) and 2.78% of NEWs (95% CI: 0.07–14.5, *p* < 0.001***) testing positive for *Arsenophonus*. In contrast, adult life stages showed high incidences of *Arsenophonus*, with the bacterium detectable in 54.5% of drones (95% CI: 32.2–75.6, *p* = 0.0198*) and 82.2% of workers (95% CI: 67.9–91.0). Workers isolated from the colony since emergence also did not develop *Arsenophonus* association on reaching forager age (95% CI: 0.00–0.06, *p* < 0.001***).

**Fig. 7:**
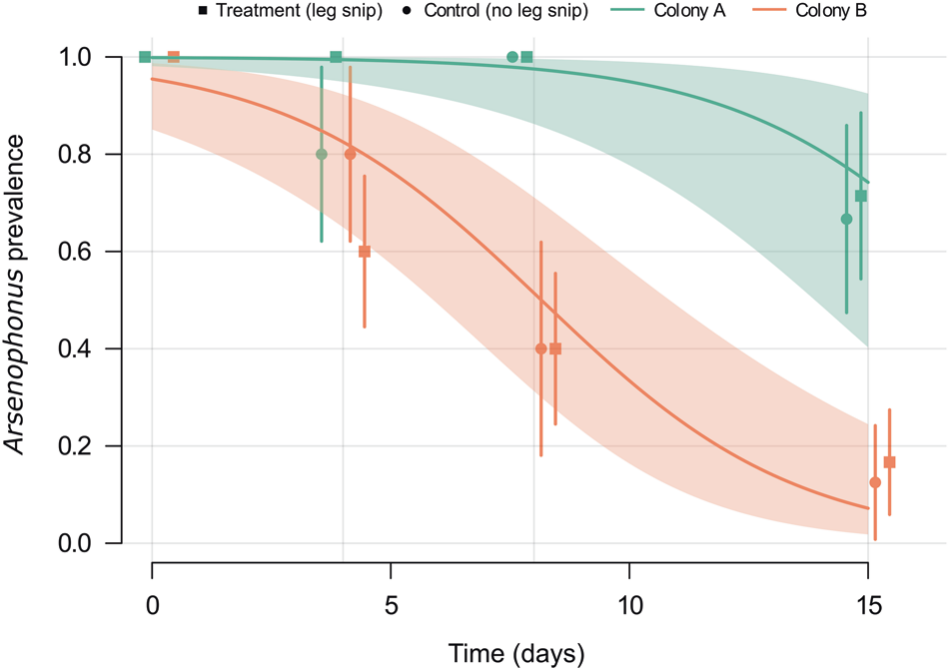
Loss of *Arsenophonus* in individuals removed from the colony and foraging environment. Binomial GLMM predictions (with 95% CI) show the proportion of individual honey bees that remain infected with *Arsenophonus* over time when removed from colonies and detained under lab conditions. The initial *Arsenophonus* status of each bee (*n* = 76) was determined by a leg snip (treatment group). An additional group of bees (*n* = 40) from the same colonies did not undergo leg snip (control group), and thus *Arsenophonus* starting prevalence was unknown. Overall predictions are shown for colony A (green) and colony B (orange), and the proportion of honey bees infected at days 0, 4, 8, and 15 is plotted independently for each colony and treatment (Color figure online).

The maintenance of *Arsenophonus* in worker bees in the absence of sources of infection was then tested (Fig. 7). Bees were removed from two infected colonies (colony A and colony B) and maintained in the laboratory for 15 days with access only to sterile food. At day 0 all individuals in the leg snip group were confirmed positive for *Arsenophonus*. Individuals in the ‘no snip control’ group were presumed, from identical colony origin, to be infected at a similar prevalence. There was no significant effect of treatment (leg snip or no snip) on the proportion of bees infected with *Arsenophonus* at each time point (LRT: *X*^2^ = 0.253, df = 1, *p* = 0.615), suggesting the leg snip did not impact our results. Overall, the proportion of bees infected with *Arsenophonus* decreased significantly over time (*p* < 0.001***) indicating loss of the bacterium. However, variation was evident at a colony level, and a significant effect of colony was observed (LRT: *X*^2^ = 20.2, df = 1, *p* < 0.001***). Colony B lost *Arsenophonus* at a significantly faster rate (*p* < 0.001***), with fewer than 20% of individuals infected by day 15. Control bees taken from uninfected colonies maintained 0% infection throughout the study. See Supplementary information Table 3 for a full summary.

**Fig. 8:**
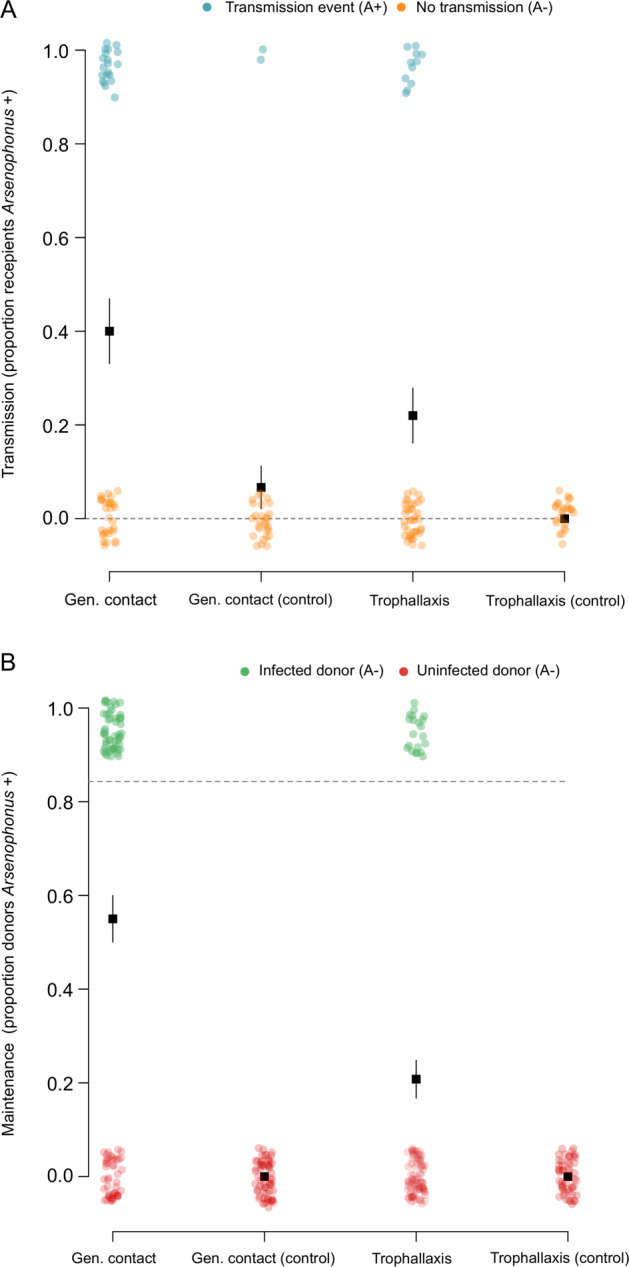
Horizontal transmission and maintenance of *Arsenophonus* in honey bees under two social conditions. **A** Uninfected (recipient) bees were mixed with (**B**) infected (donor) bees and allowed either general contact or contact via trophallaxis only. For control groups, recipients were mixed with uninfected bees. Dotted lines indicate the prevalence of *Arsenophonus* in recipients and donors at the start of the transmission period. Note, all control bees started uninfected (*Arsenophous* prevalence = 0%). After 5 days of social interaction the *Arsenophonus* status of recipients (**A**) and donors (**B**) is shown. Transmission to recipients occurred under general contact and trophallaxis, but at varying levels. Recipients mixed with uninfected (control) bees did not acquire *Arsenophonus*, with the exception of two individuals in the general contact treatment. Coloured dots show raw binomial data jittered (0 = *Arsenophonus−*, 1 = *Arsenophonus*+) and error bars indicate binomial SE. Each group was replicated (*n* = 10), with five donor bees and ten recipient bees per replicate.

Horizontal acquisition of *Arsenophonus* was tested in a separate experiment, where uninfected recipient bees were exposed to infected donor bees, either through trophallaxis or general contact. Acquisition of *Arsenophonus* among recipient bees was observed under both of these social conditions (Fig. 8A). The rate of *Arsenophonus* gain for recipients was higher under general contact exposure (recipients, 40.0%, 95% CI: 26.4–54.8) compared to trophallaxis (recipients, 22.0%, 95% CI: 11.5–36.0) and social context had a significant effect in the model (GLMM, LRT: *X*^2^ = 5.22, df = 1, *p* = 0.0223*).

Within this experiment, a subset of donors lost the infection from the 85% prevalence starting threshold (Fig. 8B), paralleling the previous observation that infection was unstable under laboratory isolation. Horizontal acquisition between donor and recipient bees contrasted to control pots where the same contact was with uninfected ‘donor’ bees. Here, recipient controls under trophallaxis remained uninfected (control recipients, 0%, 95% CI: 0–13.7), however two control recipients under general contact tested positive for *Arsenophonus* (control recipients, 6.66%, 95% CI: 0.818–22.1). The fate of individuals (live/dead at end of transmission period) emerged as an important predictor of infection status (GLMM, LRT: *X*^2^ = 7.64, df = 1, *p* = 0.00571**), with individuals that had died during the study more commonly associated with *Arsenophonus* infection (see Supplementary information Fig. 6 and Supplementary information Table 4 for a full summary).

## Discussion

The diversity of existing symbiotic interactions represents the result of historical evolutionary transitions between lifestyles. Here, we investigate the symbiotic lifestyle of *Arsenophonus* associated with honey bees. A significant contingent of the *Arsenophonus* clade is in the latter stages of an evolutionary trajectory towards obligate intracellular life [30–32], and until now all characterised members of the genus demonstrate a heritable lifestyle, at least to some degree [26, 27]. We here show that *Arsenophonus* associated with honey bees deviates significantly from this heritable model, instead demonstrating strong seasonal patterns and a dynamic association that appears to be driven by social transmission with an additional, unidentified, environmental reservoir. These results shed new light on evolutionary transitions and symbiotic diversity within a clade of heritable arthropod symbionts.

We have identified the *Arsenophonus*–honey bee strain as the first within the clade to show little evidence of heritability, with evidence for this coming from multiple sources. First, the absence of the bacterium in eggs of infected colonies corroborates previous findings that *Arsenophonus* is not transovarially transmitted in honey bees [43], while very low prevalence in early host life stages provides new evidence that no alternative routes of VT are operating in the association. Secondly, the pronounced seasonal dynamics we observed for *Arsenophonus* are not consistent with the epidemiology of heritable symbionts, whose infection prevalence generally remains relatively static within a host generation though may vary dynamically between generations [70]. Within these dynamics, colonies appear to lose *Arsenophonus* over the winter, or the association declines to a level undetectable by our survey. Finally, experiments show first that workers bees with *Arsenophonus* infection lose it under laboratory conditions under social isolation, and also that social contact can result in acquisition of infection. Overall, *Arsenophonus* presents as a horizontally acquired infection of honey bees rather than a persistent VT transmitted one.

This acquisition-loss cycle occurs for a bacterium that is otherwise nested into a clade of heritable microbes, with phylogenomic analysis indicating *Arsenophonus* from bees lies in the same subclade as the male-killing microbe, *Arsenophonus nasoniae*. Whilst predominantly associated with the parasitic *Nasonia* wasp, *A. nasoniae* is an extracellular symbiont that also has phases outside of the wasp host [71], is culturable [28], and is also maintained by a combination of vertical and infectious transmission [13]. *Arsenophonus nasoniae* and the bee *Arsenophonus* also share the capacity to establish through the gut [71], indicating this pathway is preserved in the clade. The genome of *Arsenophonus* from the honey bee is, like *A. nasoniae*, relatively large in size and not subject to the degradation process that occurs in the obligate symbionts in the genus. The two strains have markedly similar predicted metabolic competence profiles, which further supports the likely culturability of the *Arsenophonus* from *Apis*, and its capacity to survive and replicate outside of the host environment. Taken together, the *A. nasoniae*/honey bee *Arsenophonus* clade is most parsimoniously regarded as a retained free-living, insect-associated microbe in a clade that has evolved into endosymbiosis, and indeed obligate endosymbiosis, in other cases. The alternate model—reacquisition of free-living capacity—is less probable as this would require the re-establishment of multiple genetic systems allowing growth outside of a host environment. This scenario is not, however, impossible, as previous work on *Coxiella* indicated the striking emergence of an infectious vertebrate pathogen from a clade of heritable, and sometimes obligate, endosymbionts of arthropods [22].

Our data thus indicate that the clade *Arsenophonus* contains more transmission diversity than previously considered. Knowledge of the selective forces that drive the emergence of symbiotic diversity is imperative for understanding important transitions such as the emergence of pathogenic agents from commensal partners, and vice versa [21, 22, 72]. Enterobacteriaceae genera appear to be well adapted for transitioning between ecological and symbiotic niches [73, 74]; for example, *Pantoea*, *Sodalis* and *Serratia* include notable symbionts of insects [75–77] but are largely comprised of representatives from soil, plants and clinical settings [21, 74, 77]. Likewise, the genus *Photorhabdus* includes both defensive symbionts and environmentally acquired pathogens of invertebrates [78, 79], but also a primary pathogen of humans with an unidentified transmission route [80, 81]. Among Enterobacteriaceae, the *Arsenophonus* genus differs, in that all strains to date are believed to be arthropod host-restricted and previously environmental acquisition has not been shown to occur routinely [27].

Given that vertical transmission is not operating in the honey bee–*Arsenophonus* association, the questions arises: what are the main drivers of HT? Do honey bees acquire infection predominantly from infected conspecifics, or are infections acquired from the wider environment (e.g. additional host species or reservoirs)? While the symbiotic phenotype of the honey bee–*Arsenophonus* remains unknown, we identified horizontal (infectious) transmission when general contact between conspecifics was allowed, and when contact was via trophallaxis exclusively. For a heritable symbiont, maintenance or reversal to the ancestral (horizontal) transmission route has a clear adaptive benefit in a eusocial host, typified by high host density and low genetic diversity [82], and social acquisition can prevent workers being the evolutionary dead ends they are often considered for heritable symbionts [83]. Localisation in the gut and detection in faeces is consistent with a faecal—oral route, which could allow effective social transmission within a colony and to other hosts via shared floral resources [84–86]. Indeed, *Arsenophonus* has previously been detected on flowers [87], but until now direct evidence for the potential capacity to transmit via this route was lacking.

The marked seasonal incidence of *Arsenophonus*, peaking in autumn, has a number of potential drivers. Shedding and accumulation of the bacterium in the foraging environment (e.g. on flowers) may generate increased infection risk as the foraging season proceeds, as observed for some parasite species [88]. However, this would be contingent on the survival of *Arsenophonus* in the environment, a trait conventionally considered absent from this class of insect symbionts, despite relatively large symbiont genomes and cell free cultivability found within the clade [89, 90]. Alternatively, but not mutually exclusively, *Arsenophonus* dynamics may reflect the activity period of an environmental reservoir or additional host species that is responsible for transmission to honey bees. Comparable seasonality is observed for *Spiroplasma* in honey bees, with peak prevalence of *S. melliferum* aligning with peak flowering periods [91, 92]. This latter hypothesis may explain our observation that apiary is a significant predictor of a colonies *Arsenophonus* status, while over broad spatial scales infection prevalence does not vary notably. Here, localised spatial pattern reflects shared exposure to reservoirs of infection, with colonies originating from the same apiary often overlapping in foraging area [93]. Relevant here are reports of *Arsenophonus* associated with pollen and nectaring sites [42, 87], providing further evidence for environmental transmission [87]. Other processes, such as drifting of infected bees within apiaries, could drive inter-colonial transmission of *Arsenophonus* and feedback to drive the localised infection prevalence we observed.

The position of the honey bee *Arsenophonus* on the parasitism-mutualism continuum remains speculative. Our observations of HT, systemic infections and a higher prevalence among dead hosts strengthens interpretations of previous work correlating *Arsenophonus* with poor health outcomes in bees [44, 45, 94]. HT is considered to increase the scope for the evolution of virulence [17, 18], although this trait alone is insufficient to draw conclusions regarding symbiont phenotype, as many avirulent or beneficial microbes are transmitted horizontally [15, 95]. The association with dead hosts may implicate *Arsenophonus* directly or represent opportunistic proliferation in a compromised host. Alternatively, saprophytic growth on cadavers may be occurring, a capacity demonstrated by *A. nasoniae* in fly puparia [96]. Further work is needed to determine if health outcomes are causal in honey bees and to characterise the transmission phenotype of *Arsenophonus* reported from other bee species [94, 97–99].

To conclude, the honey bee–*Arsenophonus* contrasts in its phenotype from the rest of the heritable clade, as vertical transmission is not the predominant transmission mode. Instead, HT occurs via social interactions, colonisation can occur in the gut and environmental reservoirs appear to be necessary for maintenance of the association. Our data establish the honey bee–*Arsenophonus* as a key link in a symbiont clades trajectory from free-living ancestral life to obligate mutualism threatened by mutational decay. This will provide new avenues for research into the emergence of symbiotic diversity.

## Supplementary information

Supplementary Information

## Data Availability

All data associated with this study can be publicly accessed at 10.6084/m9.figshare.c.5073479.v1.
