# Supplementary material for: Transitions in symbiosis: evidence for environmental acquisition and social transmission within a clade of heritable symbionts
Source: ISME J. 2021 May 3;15(10):2956–68. doi: 10.1038/s41396-021-00977-z (PMC8443716; doi:10.1038/s41396-021-00977-z)
Supplement: Supplementary file 1 — Supplementary Information [file 41396_2021_977_MOESM1_ESM.pdf]

Supplementary Information

Transitions in symbiosis: evidence for environmental acquisition & social transmission within a clade of heritable symbionts

Georgia C Drew[1,3], Giles E Budge[2], Crystal L Frost[3], Peter Neumann[4], Stefanos Siozios[3], Orlando Yañez[4] & Gregory DD Hurst[3]

[1]Department of Zoology, University of Oxford, Oxford, UK

[2]School of Natural and Environmental Sciences, Newcastle University, Newcastle upon Tyne, UK

[3]Institute of Integrative Biology, University of Liverpool, Liverpool, UK

[4]Institute of Bee Health, Vetsuisse Faculty, University of Bern, Bern, Switzerland

Corresponding author: georgia.drew@zoo.ox.ac.uk

Supplementary Methods

Genomic analysis

In total, 53 core ribosomal protein coding genes were extracted from *Arsenophonus* from honey bees and compared to 12 available *Arsenophonus* genomes with the free-living species *Proteus mirabilis* and *Providencia stuartii* used as outgroups (see SI Table 1). Relatedness of strains was estimated by Bayesian inference on the bases of the concatenated alignment of the 53 ribosomal protein dataset, completed using Phylobayes-MPI [1] and the CAT-GTR model. Two independent chains were run in parallel for over 25,000 cycles each until convergence occurred (maxdiff < 0.1). A second phylogenetic analysis was performed on the concatenated set of 155 single-copy core orthologous proteins determined using OrthoFinder v2.3.11 [2]. In this case a maximum likelihood (ML) phylogenetic inference was conducted with IQ-TREE v2.0.3 [3] and the JTT+F+R3 model

selected using ModelFinder [4]. Robustness was assessed using the ultrafast bootstrap approximation method as implemented in IQ-TREE and 1,000 replicates [5].

The metabolic potential of the *Arsenophonus* genome from honey bees was evaluated by computing the KEGG module completion ratio (MCR) using Genomaple-2.3.2 server [6] and compared to other *Arsenophonus* strains as well as free-living (*Proteus mirabilis*, *Providencia stuartii* and *Providencia rettgeri*) or symbiotic (*Candidatus* Riesia pediculicola) relatives. In addition, the predicted protein sequences were grouped into Clusters of Orthologous Groups (COG) functional categories using the eggNOG 5.0 database [7]. Contigs of putative phage origin were identified using the PHAge Search Tool Enhanced Release (PHASTER) web server based on sequence similarities searches. Finally, the synteny between the *Arsenophonus* genome from honey bees and the close relative *Arsenophonus nasoniae* strain was assessed using D-Geneis [8] and minimap2 [9]. Data visualisation was performed in R v3.6.3 [10] using ggplot2 package [11] while phylogenetic trees were prepared using the EvolView 3 [12].

SI Tab. 1 *Arsenophonus* genomes analysed for phylogenomics & comparative genomics

Strain	Accession ID	Bases	Status
Arsenophonus of *Apis mellifera*	This study	3315916	draft
Arsenophonus of *Aphis craccivora*	GCF_013460135.1	2424437	complete
Arsenophonus of *Bemisia tabaci* Asia II 3	GCF_004118055.1	2328823	draft
Arsenophonus of *Bemisia tabaci* Q2	GCA_902713415.1	1860497	draft
Arsenophonus of *Aleurodicus dispersus*	LR025108.1	663125	complete
Arsenophonus of *Aleurodicus floccissimus*	GCF_900343025.1	3001875	draft
Arsenophonus nasoniae (DSM15247)	AUCC01000000	3670548	draft
Arsenophonus of *Entylia carinata*	NHNG00000000.1	3228533	draft
Arsenophonus of *Nilaparvata lugens*	GCF_000757905.1	2953863	draft
Arsenophonus of *Lipoptena fortisetosa*	CP013920.1	836724	complete
Candidatus Arsenophonus triatominarum	LWMI00000000	3858720	draft
Arsenophonus nasoniae (FIN)	GCA_004768525.1	5262212	complete
Arsenophonus melophagi	http://users.prf.jcu.cz/novake01/	1155312	draft

Honey bee maintenance & handling (laboratory conditions)

Unless otherwise stated, under laboratory conditions Western honey bees were maintained on filter paper in plastic deli pot cages at 32°C ± 2 °C and incubators were kept dark. Ambrosia bee fondant and 50% sucrose solution were fed *ad libitum.* Where experiments required immobilisation of honey bees (e.g. for removal of tarsus tissue) this was done at 4°C. Marking of bees was done using queen paint.

DNA extraction by Promega Wizard purification

A Promega Wizard genomic DNA purification kit with a protocol modified for insects was used to extract DNA from honey bee samples. Samples were homogenised in nuclei lysis solution (250μl) and incubated for 30 min at 65°C. Protein precipitation solution (80μl) was added and samples were vortexed and held on ice (5 min). Samples were centrifuged (14,725 rpm, 4 min) and supernatant mixed with filtered isopropanol (150μl) via inversion. After further centrifugation (14,725 rpm, 2 min), supernatants were discarded and 70% EtOH (150μl) added to pellets, before gentle vortexing and centrifugation (14,800rpm, 1 min). Supernatants were discarded and pellets airdried (65°C, 30 min) before resuspension in molecular grade H_20 (60μl) at 4°C overnight.

PCR cycling & sequencing conditions

Polymerase chain reaction assays (PCR) were based on a total volume of 15μl, containing GoTaq Hot Start Green Master Mix (7.5μl), nuclease free water (5.5μl), forward and reverse 10μM primer (0.5μl) and template DNA (1μl). PCR amplifications were performed on Applied Biosystem Veriti cycler under the following conditions: 95°C for 2 min, 35 cycles at 94°C for 30 s, 58°C for 30 s (variable, depending on primer Tm) 72°C for 1 min with a final extension of 72°C for 10 min. Primers used for detection of *Arsenophonus* targeted *fbaA* (5' -GCCGCTAAGGTTGGTTCTCC – 3' and 5' - CCTGAACCACCATGGAAAACAAAA – 3') and were adapted from a previous study [13]. Products were visualised on 1.5% w/v agarose gel with 0.5μg/mL ethidium bromide (110 v for 50 min) Every run included a positive, negative and no template control. Where samples were to be sequenced, PCR products were cleaned of unincorporated primers and nucleotides using 0.2μl of SAP (shrimp alkaline phosphatase), 0.05μl of Exonuclease I, 0.7μl of 10X RX Buffer and 1.05μl of molecular grade H_20 and incubated at 37°C for 45 min, followed by 80°C for 15 min. Purified products were Sanger sequenced through both strands in house on an ABI Prism 3010x or outsourced to GATC Biotech.

Validation of *Arsenophonus* PCR assay sensitivity

Sensitivity of the detection method for *Arsenophonus* was assessed by serial dilution of *Arsenophonus* template with uninfected honey bee template DNA. In all tested cases (N = 6)

Arsenophonus DNA remained detectable at 10^{-1} and 10^{-2} dilutions, in 4/6 cases *Arsenophonus* was detectable at 10^{-3} and 10^{-4} dilutions of honey bee template, providing confidence in the sensitivity of our detection method.

Localisation of *Arsenophonus* within the gut

To visualise *Arsenophonus* using fluorescence in situ hybridization (FISH), whole guts were dissected from live worker bees (chilled on ice) and placed into Carnoy's fixative (60% EtOH, 30% chloroform, 10% acetic acid) for 24–48hrs, washed with 100% EtOH (x 3) and incubated with hybridization buffer (20Mm Tris-HCL, 0.9M NaCl, 0.01% SDS, 30% formamide, 100 pmol/ml probe) at room temperature in the dark (~15 hrs). The symbiont was targeted using an *Arsenophonus* specific probe (TCATGACCACAACCTCCA) [14] with a 5' Alexa Fluor 647 fluorochrome. Tissues were washed with pre-heated (~48°C) buffer (20Mm Tris-HCL, 0.9M NaCl, 0.01% SDS, 0.05M EDTA) x 3 for 10 min (room temperature, dark) and mounted on glass slides with DAPI containing ProLong Diamond anti-fade (Fisher Scientific).

Selection criteria for colonies used in infection maintenance & social transmission experiments

As the *Arsenophonus* status of individual bees included in the infection maintenance and social transmission experiment could not be pre-determined (demands destructive sampling), we selected individuals from colonies with a threshold *Arsenophonus* prevalence of 85%. For each colony this was determined by screens of 15 worker bees individually. Samples were washed in sterile H_20 and exposed to ultra violet (UV) light for 10 minutes to cross link contaminating external DNA. DNA was extracted using a Promega Wizard genomic DNA purification kit with a protocol modified for insects and screened for *Arsenophonus* using PCR assays (as described above). Workers used in negative control assays were from colonies where no adult worker had returned a positive result for *Arsenophonus* detection. For the social transmission study, a sample of NEWs (although generally considered sterile [15] and rarely associated with *Arsenophonus*, see main text Figure 6) were similarly screened to ensure *Arsenophonus* was not present prior to mixing with infected conspecifics. For the transmission experiment, 85% and 0% were thus taken as a proxy starting prevalence (dotted lines on main text Figure 8) for workers and NEWs respectively.

Supplementary Results

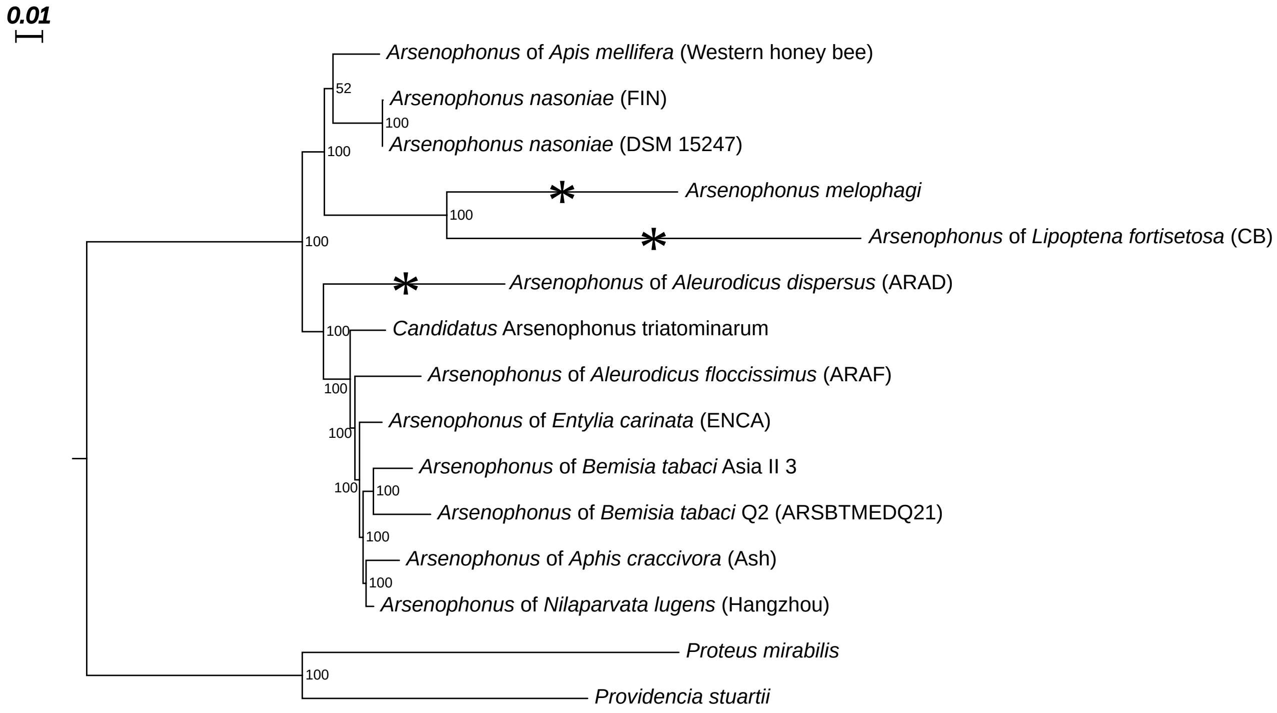

SI Fig. 1 *Arsenophonus* phylogeny based on 115 single-copy core gene. Both phylogenomic reconstructions using the set of 53 ribosomal proteins (main text) or a set of single-copy core genes (this figure) supported the position of the honey bee *Arsenophonus* in the wider clade and its relation to the son-killer species *Arsenophonus nasoniae*

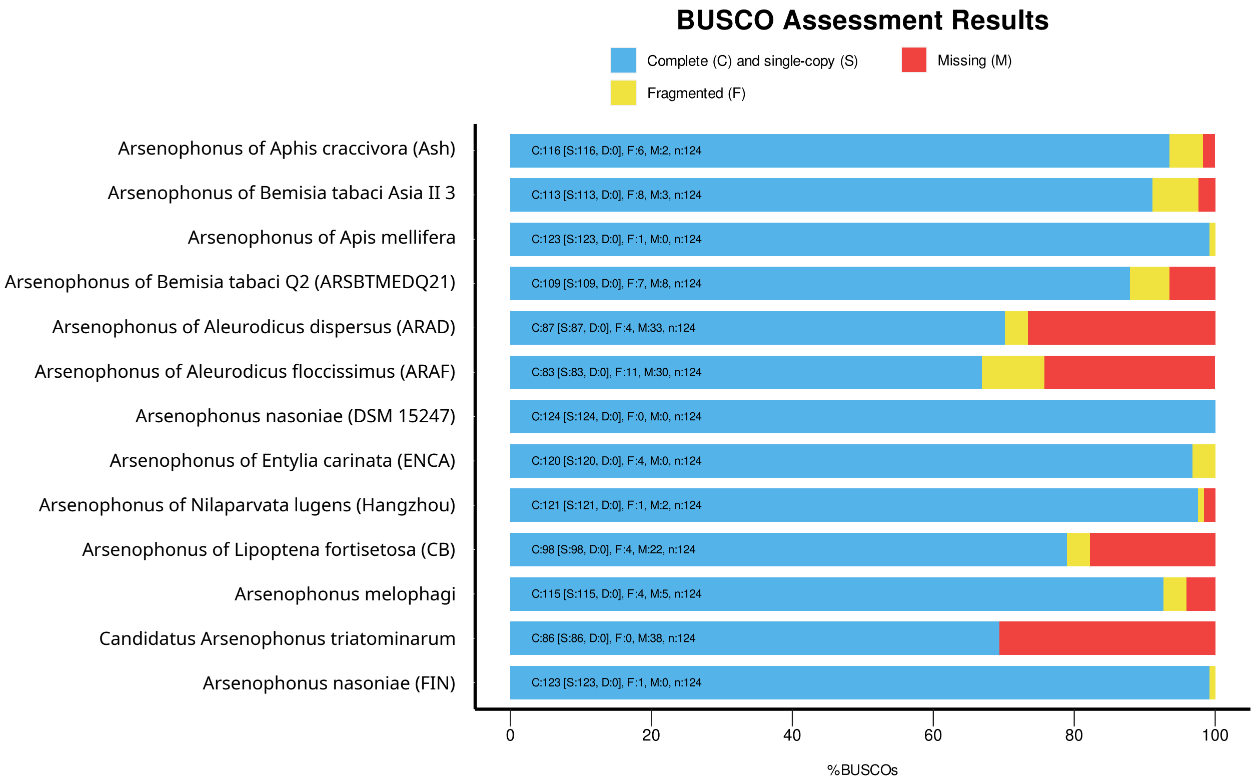

SI Fig.2 Assessment of genome assembly for *Arsenophonus* of *Apis mellifera*. BUSCO assessment of genome assembly completeness for *Arsenophonus* from *Apis mellifera* (this study) suggest a near complete genome. BUSCO scores for twelve other available *Arsenophonus* genomes (see SI Tab.1) are shown alongside for comparison.

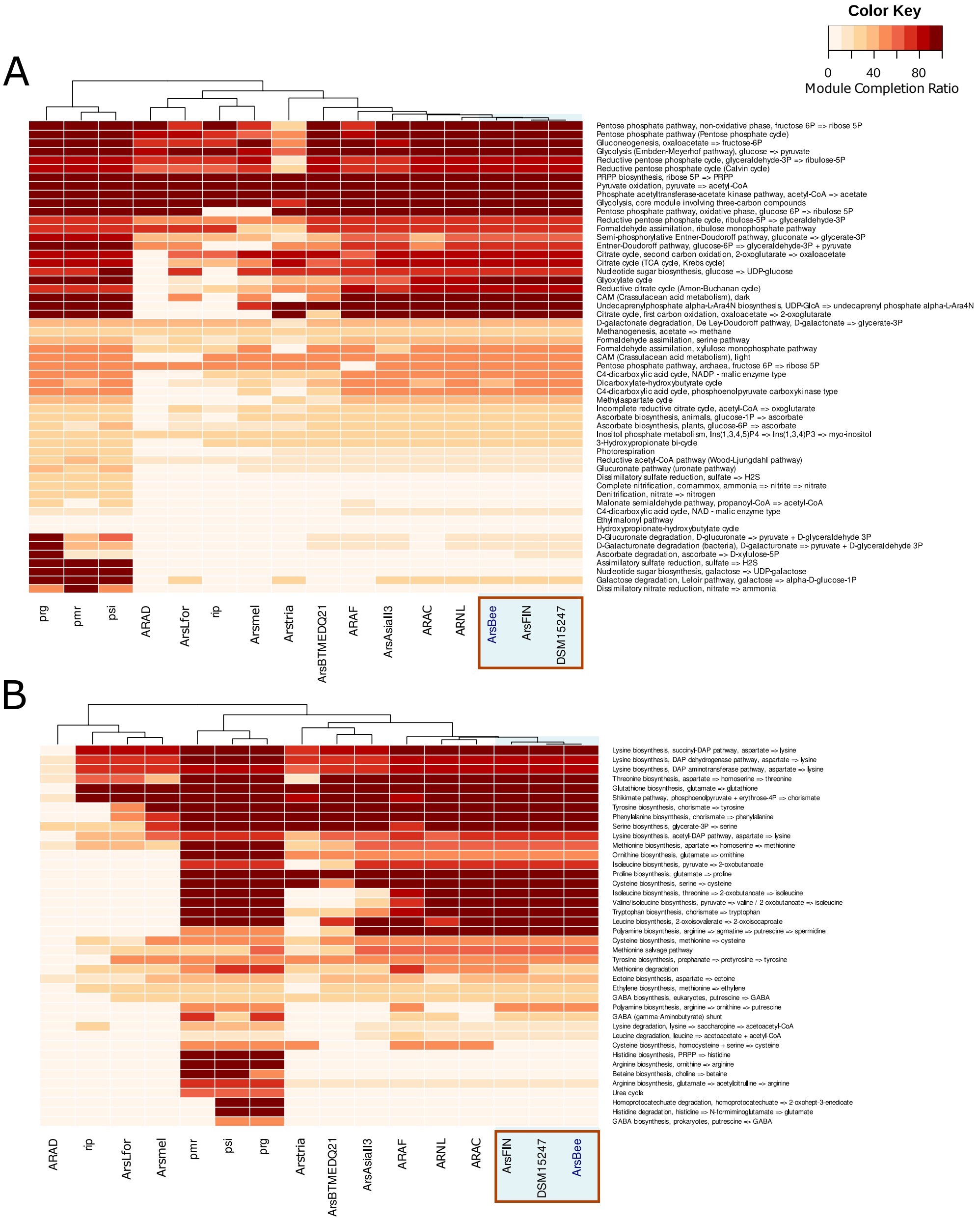

SI Fig.3 The metabolic potential of *Arsenophonus* strains and comparison to free-living or symbiotic relatives. (A) Carbohydrate and energy metabolism and (B) amino acid metabolism**.** The module completion ratio (MCR) for each KEGG functional category was calculated using Genomaple-2.3.2. Dendrograms represent hierarchical clustering of the analysed microbial genomes based on the MCRs values. The clustering of ArsBeeCH strain with previously sequenced *Arsenophonus nasoniae* strains is highlighted. pmr: *Proteus mirabilis* HI4320, psi: *Providencia stuartii* MRSN 2154, prg: *Providencia rettgeri* RB151, rip: *Candidatus* Riesia pediculicola, ArsFIN: *Arsenophonus nasoniae* (FIN), ArsBeeCH: *Arsenophonus* of *Apis mellifera*, DSM15247: *Arsenophonus nasoniae* (DSM15247), Arsmel: *Arsenophonus melophagi*, ArsLfor: *Arsenophonus* of *Lipoptena fortisetosa*, ARAD; *Arsenophonus* of *Aleurodicus dispersus*, Arstria: *Candidatus* Arsenophonus triatominarum, ARAF: *Arsenophonus* of *Aleurodicus floccissimus*, ArsAsiaII3: *Arsenophonus* of *Bemisia tabaci* Asia II 3, ArsBTMEDQ21: Arsenophonus of Bemisia tabaci Q2, ARAC: *Arsenophonus* of *Aphis craccivora*, ARNL: *Arsenophonus* of *Nilaparvata lugens*. Due to its highly fragmented status (818 contigs) the genome of *Arsenophonus* of *Entylia carinata* (ENCA) was excluded from this analysis.

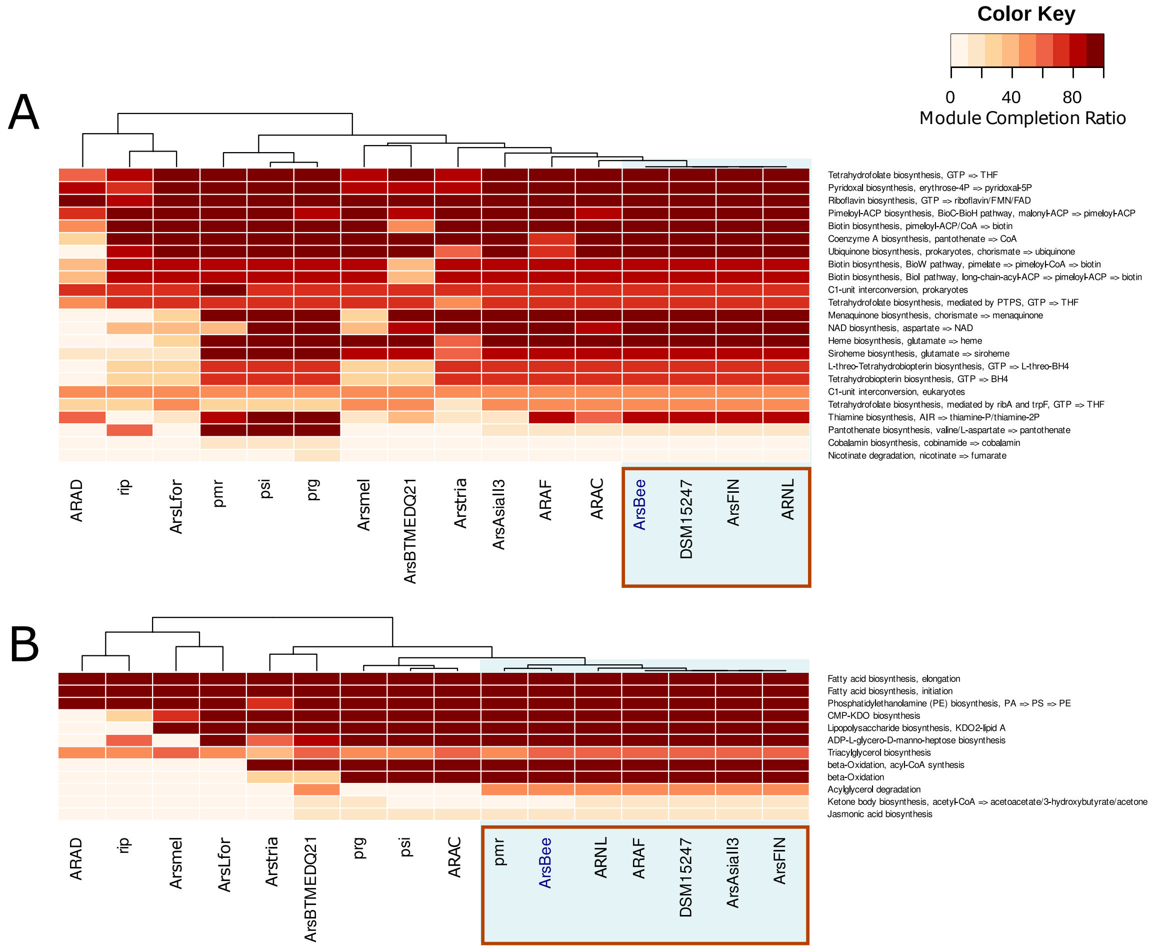

SI Fig.4 The metabolic potential of *Arsenophonus* strains and comparison to free-living or symbiotic relatives. (A) Metabolism of cofactors and vitamins and (B) lipid and glycan metabolism**.** The module completion ratio (MCR) for each KEGG functional category was calculated using Genomaple-2.3.2. Dendrograms represent hierarchical clustering of the analysed microbial genomes based on the MCRs values. The clustering of ArsBeeCH strain with previously sequenced *Arsenophonus nasoniae* strains is highlighted. pmr: *Proteus mirabilis* HI4320, psi: *Providencia stuartii* MRSN 2154, prg: *Providencia rettgeri* RB151, rip: *Candidatus* Riesia pediculicola, ArsFIN: *Arsenophonus nasoniae* (FIN), ArsBeeCH: *Arsenophonus* of *Apis mellifera*, DSM15247: *Arsenophonus nasoniae* (DSM15247), Arsmel: *Arsenophonus melophagi*, ArsLfor: *Arsenophonus* of *Lipoptena fortisetosa*, ARAD; *Arsenophonus* of *Aleurodicus dispersus*, Arstria: *Candidatus* Arsenophonus triatominarum, ARAF: *Arsenophonus* of *Aleurodicus floccissimus*, ArsAsiaII3: *Arsenophonus* of *Bemisia tabaci* Asia II 3, ArsBTMEDQ21: Arsenophonus of Bemisia tabaci Q2, ARAC: *Arsenophonus* of *Aphis craccivora*, ARNL: *Arsenophonus* of *Nilaparvata lugens*. Due to its highly fragmented status (818 contigs) the genome of *Arsenophonus* of *Entylia carinata* (ENCA) was excluded from this analysis.

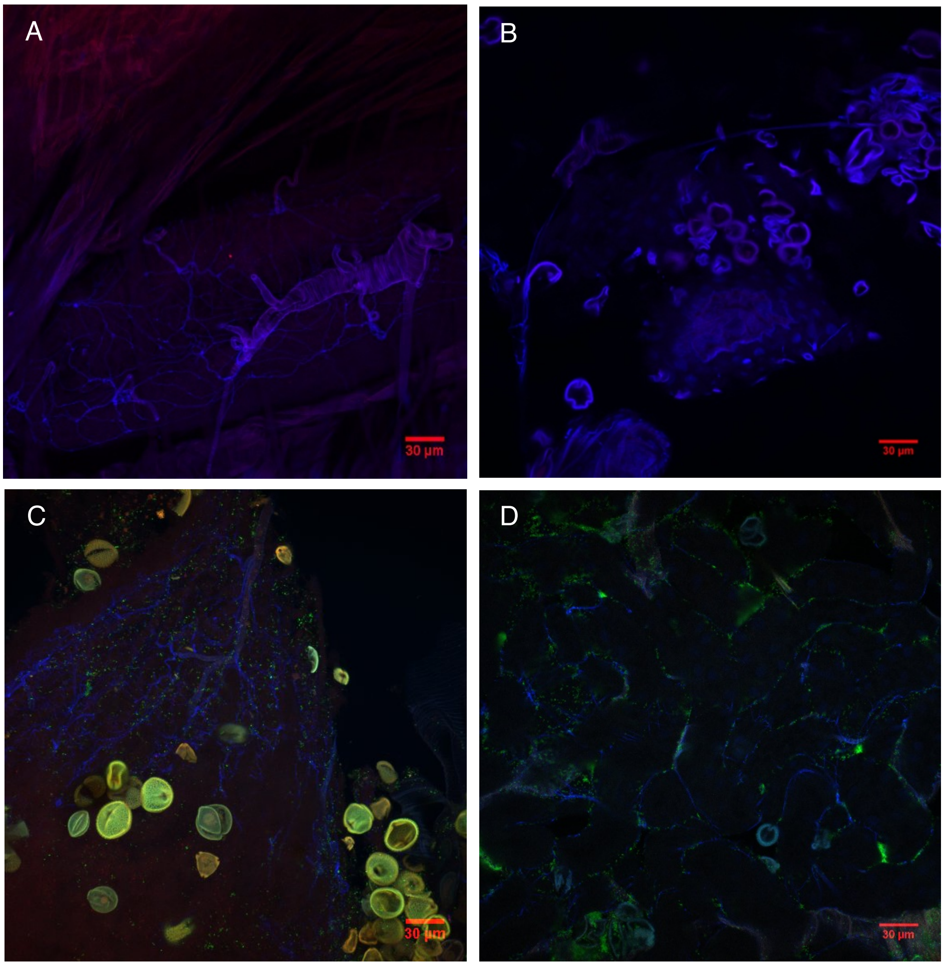

SI Fig. 5 Confocal microscopy images of whole mounted honey bee guts from colonies with no evidence of *Arsenophonus* association. Negative control mages were obtained using whole gut mounts from *Arsenophonus* negative honey bee workers. These visualisations were used to assess if signals from tissue autofluorescence or inadequate washing would generate non-specific signals from the *Arsenophonus* specific probe (red fluorescence). The absence of significant red signal suggests this was not of concern. Honey bee whole guts (A, B, C, D) were counterstained with DAPI (blue fluorescence) and symbiont DNA targeted by an *Arsenophonus* specific probe (TCATGACCACAACCTCCA) [14] 5' labelled with a Alexa Fluor 647 fluorochrome (red fluorescence, not visible). A universal bacterial FISH probe is shown highlighting other bacterial members of the gut in green fluorescence (C & D only) (TGCTGCCTCCCGTAGGA) [16], 5' tagged with a Alexa Fluor 555 fluorochrome. Images were obtained using a ZEISS LSM 880 confocal microscope with a x 40 objective and processed using Image J [17].

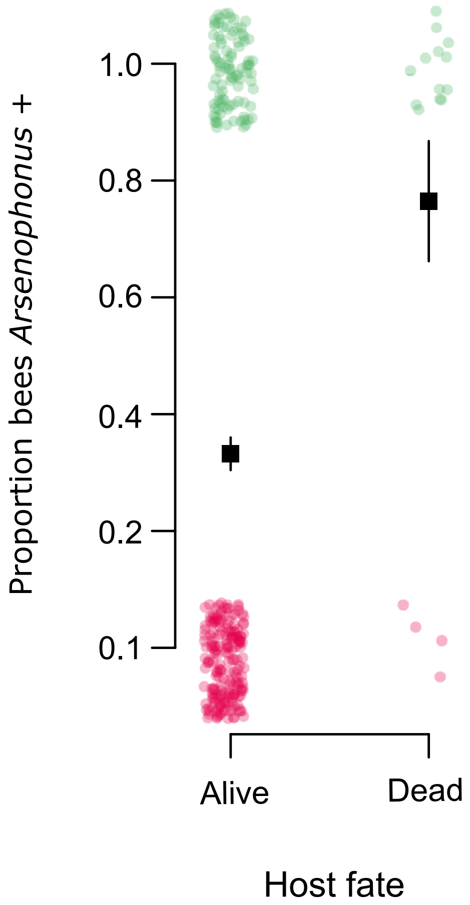

SI Fig. 6 Association between *Arsenophonus* status and honey bee fate. *Arsenophonus* prevalence was higher among individuals that died (n=17), compared to those that remained alive (n=283), during the course of the horizontal transmission study. Pink dots (0 = individual *Arsenophonus* -) and green dots (1 = individual *Arsenophonus* +). Errors bars indicate binomial SE.

Selection tables & parameter estimates from statistical models

SI Tab. 2 Model selection & parameter estimates for spatial & seasonal dynamics of *Arsenophonus* in honey bee colonies

**Model structure ^{GLMM}**	***df***	**AIC**	**Loglik**	**Dev.**
Arsenophonus status ~ Day of season^3 + (Year) + (County/Apiary/Colony.ID)	8	256.7	-120.4	240.7
Arsenophonus status ~ Day of season^3 + (Year) + (Apiary/Colony.ID)	7	254.7	-120.4	240.7
Arsenophonus status ~ Day of season^3 + (Year) + (Apiary) ^{SM}	6	252.7	-120.4	240.7
Arsenophonus status ~ Day of season^3 + (Apiary)	5	254.6	-122.3	244.6
Arsenophonus status ~ Day of season^3 + (Year)	5	264.5	-127.3	254.5
**Fixed effect**	**Est.**	**SE**	***Z***	***P***
Intercept	-0.7423	0.4502	-1.649	0.09920
Day of season^1	17.37	4.514	3.849	0.000118***
Day of season^2	-12.26	4.162	-2.946	0.003223**
Day of season^3	-7.736	3.495	-2.213	0.02687*
**Random effect**	***N***	**Var.**	**SD**	
Apiary	45	1.155	1.075	
Year	5	0.4944	0.7031	

Note, day of the season (March 20th = day 0, November 8th = day 232) was modelled as a fixed effect 3rd order polynomial. A total of 229 observations of *Arsenophonus* status were included in the analysis. ^{SM} Selected model, ^{df} Degrees of freedom, ^{AIC} Akaike's information criterion, $^{Dev.}$ Deviance, $^{Est.}$ Coefficient estimate, $^{Var.}$ Variance. (*P* <***0.001, **0.01, *0.05)

SI Tab. 3 Model selection & parameter estimates for *Arsenophonus* loss in individuals removed from the colony & foraging environment

**Model structure** ^{GLMM}	***df***	**AIC**	**Loglik**	**Dev.**
Arsenophonus status ~ Time * Colony + Treatment + (1 | Pot)	6	100.7	-44.3	88.7
Arsenophonus status ~ Time * Colony + (1 | Pot)	5	98.94	-44.5	88.9
Arsenophonus status ~ Time + Colony + (1 | Pot) ^{SM}	4	97.52	-44.8	89.5
Arsenophonus status ~ Time + (1 | Pot)	3	115.8	-54.9	109.8
**Fixed effect**	**Est.**	**SE**	***Z***	***P***
Intercept	6.661	1.384	4.813	< 0.001***
Time	-0.3736	0.0739	-5.055	< 0.001***
Colony (Colony B)	-3.615	1.058	-3.414	< 0.001***
**Random effect**	***N***	**Var.**	**SD**	
Pot	19	0.9725	0.9862	

^{SM} Selected model, ^{df} Degrees of freedom, ^{AIC} Akaike's information criterion, $^{Dev.}$ Deviance, $^{Est.}$ Coefficient estimate, $^{Var.}$ Variance. (*P* <***0.001, **0.01, *0.05)

SI Tab.4 Horizontal transmission of *Arsenophonus* in honey bees under two social conditions

**Model structure** ^{GLMM}	***df***	**AIC**	**Loglik**	**Dev.**
Arsenophonus status ~ Bee status * Transmission + Colony + Fate + (1| Pot)	9	307.2	-144.6	289.2
Arsenophonus status ~ Bee status + Transmission + Colony + Fate + (1| Pot)	8	306.2	-145.1	290.2
Arsenophonus status ~ Bee status + Transmission + Fate + (1| Pot) ^{SM}	5	303.3	-146.7	293.4
Arsenophonus status ~ Bee status + Transmission + (1| Pot)	4	309.0	-150.5	300.9
Arsenophonus status ~ Bee status + Fate + (1| Pot)	4	306.6	-149.3	298.6
**Fixed effect**	**Est.**	**SE**	***Z***	***P***
Intercept	-1.612	0.5545	-2.907	0.00365 **
Status (NEW)	-0.5058	0.3298	-1.534	0.1251
Transmission (General contact)	1.842	0.7714	2.387	0.01697 *
Fate (Dead)	1.930	0.7385	2.613	0.00898 **
**Random effect**	***N***	**Var.**	**SD**	
Pot	20	2.371	1.54	

Bee status = Worker *or* NEW, Transmission = Gen contact *or* Trophallaxis, Fate = Dead *or* Alive. Total observations (N = 300). ^{SM} Selected model, ^{df} Degrees of freedom, ^{AIC} Akaike's information criterion, $^{Dev.}$ Deviance, $^{Est.}$ Coefficient estimate, $^{Var.}$ Variance. (*P* <***0.001, **0.01, *0.05)

Supplementary References

1. Lartillot N, Rodrigue N, Stubbs D, Richer J. PhyloBayes MPI: Phylogenetic Reconstruction with Infinite Mixtures of Profiles in a Parallel Environment. *Syst Biol* 2013; **62**: 611–615.
2. Emms DM, Kelly S. OrthoFinder: solving fundamental biases in whole genome comparisons dramatically improves orthogroup inference accuracy. *Genome Biol* 2015; **16**: 157.
3. Nguyen L-T, Schmidt HA, von Haeseler A, Minh BQ. IQ-TREE: A Fast and Effective Stochastic Algorithm for Estimating Maximum-Likelihood Phylogenies. *Mol Biol Evol* 2015; **32**: 268–274.
4. Kalyaanamoorthy S, Minh BQ, Wong TKF, von Haeseler A, Jermiin LS. ModelFinder: fast model selection for accurate phylogenetic estimates. *Nat Methods* 2017; **14**: 587–587.
5. Hoang DT, Chernomor O, von Haeseler A, Minh BQ, Vinh LS. UFBoot2: Improving the Ultrafast Bootstrap Approximation. *Mol Biol Evol* 2018; **35**: 518–522.
6. Takami H, Taniguchi T, Arai W, Takemoto K, Moriya Y, Goto S. An automated system for evaluation of the potential functionome: MAPLE version 2.1.0. *DNA Res* 2016; **23**: 467–475.
7. Huerta-Cepas J, Szklarczyk D, Heller D, Hernández-Plaza A, Forslund SK, Cook H, et al. eggNOG 5.0: a hierarchical, functionally and phylogenetically annotated orthology resource based on 5090 organisms and 2502 viruses. *Nucleic Acids Res* 2019; **47**: D309–D314.
8. Cabanettes F, Klopp C. D-GENIES: dot plot large genomes in an interactive, efficient and simple way. *PeerJ* 2018; **6**: e4958.

9. Li H. Minimap2: pairwise alignment for nucleotide sequences. *Bioinformatics* 2018; **34**: 3094–3100.
10. R Core Team. A language and environment for statistical computing. R Foundation for Statistical Computing, Vienna, Austria. URL http://www.R-project.org/. *HttpwwwR-Proj* 2013; 2013–2013.
11. Wickham H. ggplot2: Elegant Graphics for Data Analysis. 2009. Springer-Verlag New York.
12. Subramanian B, Gao S, Lercher MJ, Hu S, Chen W-H. Evolview v3: a webserver for visualization, annotation, and management of phylogenetic trees. *Nucleic Acids Res* 2019; **47**: W270–W275.
13. Duron O, Wilkes TE, Hurst GDD. Interspecific transmission of a male-killing bacterium on an ecological timescale. *Ecol Lett* 2010; **13**: 1139–1148.
14. Gottlieb Y, Ghanim M, Gueguen G, Kontsedalov S, Vavre F, Fleury F, et al. Inherited intracellular ecosystem: symbiotic bacteria share bacteriocytes in whiteflies. *FASEB J* 2008; **22**: 2591–2599.
15. Gilliam M. Microbial Sterility of the Intestinal Content of the Immature Honey Bee, Apis mellifera1,2,3. *Ann Entomol Soc Am* 1971; **64**: 315–316.
16. Martinson VG, Moy J, Moran NA. Establishment of characteristic gut bacteria during development of the honeybee worker. *Appl Environ Microbiol* 2012; **78**: 2830–2840.
17. Schneider CA, Rasband WS, Eliceiri KW. NIH Image to ImageJ: 25 years of image analysis. *Nat Methods* 2012; **9**: 671–675.
